# Polydatin Prevents Lipopolysaccharide (LPS)-Induced Parkinson's Disease via Regulation of the AKT/GSK3β-Nrf2/NF-κB Signaling Axis

**DOI:** 10.3389/fimmu.2018.02527

**Published:** 2018-11-05

**Authors:** Bingxu Huang, Juxiong Liu, Tianyu Meng, Yuhang Li, Dewei He, Xin Ran, Guangxin Chen, Wenjin Guo, Xingchi Kan, Shoupeng Fu, Wei Wang, Dianfeng Liu

**Affiliations:** ^1^Department of Basic Veterinary Medicine, College of Animal Science and Veterinary Medicine, Jilin University, Changchun, China; ^2^Department of Food Quality and Safety, College of Food Science and Engineering, Jilin University, Changchun, China; ^3^Department of Infection and Immunology, Institutes of Biomedical Sciences, Shanxi University, Taiyuan, China

**Keywords:** parkinson's disease, neuroinflammation, polydatin, microglia, neuroprotection

## Abstract

Parkinson's disease (PD) is a common neurodegenerative disease characterized by selective loss of dopaminergic neurons in the substantia nigra (SN). Neuroinflammation induced by over-activation of microglia leads to the death of dopaminergic neurons in the pathogenesis of PD. Therefore, downregulation of microglial activation may aid in the treatment of PD. Polydatin (PLD) has been reported to pass through the blood-brain barrier and protect against motor degeneration in the SN. However, the molecular mechanisms underlying the effects of PLD in the treatment of PD remain unclear. The present study aimed to determine whether PLD protects against dopaminergic neurodegeneration by inhibiting the activation of microglia in a rat model of lipopolysaccharide (LPS)-induced PD. Our findings indicated that PLD treatment protected dopaminergic neurons and ameliorated motor dysfunction by inhibiting microglial activation and the release of pro-inflammatory mediators. Furthermore, PLD treatment significantly increased levels of p-AKT, p-GSK-3β^Ser9^, and Nrf2, and suppressed the activation of NF-κB in the SN of rats with LPS-induced PD. To further explore the neuroprotective mechanism of PLD, we investigated the effect of PLD on activated microglial BV-2 cells. Our findings indicated that PLD inhibited the production of pro-inflammatory mediators and the activation of NF-κB pathways in LPS-induced BV-2 cells. Moreover, our results indicated that PLD enhanced levels of p-AKT, p-GSK-3β^Ser9^, and Nrf2 in BV-2 cells. After BV-2 cells were pretreated with MK2206 (an inhibitor of AKT), NP-12 (an inhibitor of GSK-3β), or Brusatol (BT; an inhibitor of Nrf2), treatment with PLD suppressed the activation of NF-κB signaling pathways and the release of pro-inflammatory mediators in activated BV-2 cells via activation of the AKT/GSK3β-Nrf2 signaling axis. Taken together, our results are the first to demonstrate that PLD prevents dopaminergic neurodegeneration due to microglial activation via regulation of the AKT/GSK3β-Nrf2/NF-κB signaling axis.

## Background

Parkinson's disease (PD), which is featured by the selective death of dopaminergic neurons in the substantia nigra (SN) of midbrain, is the second most common neurodegenerative disease, affecting up to 1% of people over the age of 60 worldwide (1). PD is associated with main manifestations, including rigidity, resting tremor and postural gait disorder, and is accompanied by progressive non-motor symptoms such as cognitive impairment, mood disturbance, sleep dysfunction, gastrointestinal problems, and dysautonomia (2–4). While previous researches have proved that the pathogenesis of PD is associated with old-age factor, environmental factor, genetic factor, oxidative stress and free radical formation, accumulating evidence indicates that the neuroinflammatory response plays a critical role in the progression of PD (5, 6).

Activated microglia represents a main factor of neuroinflammation and dopaminergic neurodegeneration (7–9). Excessive activation of microglia leads to the release of several neurotoxic factors, such as interleukin-1β (IL-1β), tumor necrosis factor-α (TNF-α), interleukin-6 (IL-6), prostaglandin E2 (PGE2), and nitric oxide (NO), which contribute to dopaminergic neurodegeneration (10, 11). Therefore, inhibiting microglial activation may aid in the treatment and prevention of PD.

Lipopolysaccharide (LPS), extracted from the outer membranes of Gram-negative bacteria, can effectively activate immune cell microglial cells in the brain. Previous studies have demonstrated that intranigral injection of LPS selectively induces the death of dopaminergic neurons (12, 13). Thus, considering the relationship between neuroinflammation and PD, intranigral injection of LPS is often used to induce animal models of PD. Given the potential role of inflammation in the pathogenesis of PD, the cellular/molecular mechanisms leading to the death of dopaminergic neurons in LPS-induced models of PD must be elucidated.

The transcription factor nuclear factor-erythroid 2-related factor 2 (Nrf2) regulates basal and inducible transcription of genes encoding protective molecules against various inflammatory and oxidative stresses (14). It has been demonstrated that nuclear factor-kappa light chain enhancer of B cells (NF-κB) is a transcription factor regulated the production of proinflammatory cytokines, Nrf2-Keap1 and the NF-κB inflammatory cascade in the pathophysiology of many diseases (15, 16). Li et al. reported that the expression levels of pro-inflammatory genes (TNF-α, inducible nitric oxide synthase (iNOS), and COX-2) are higher in Nrf2-deficient mice than in control mice (17). In addition, Ganesh Yerra et al. reported that targeting of the Nrf2-NF-κB axis exerts potential therapeutic effects in diabetic neuropathy (18). Glycogen synthase kinase-3β (GSK-3β) plays an important role in downregulating the H_2_O_2_-induced oxidative stress and cell death by eliciting the transcription factor Nrf2 (19). Cuadrado et al. have demonstrated that dimethyl fumarate exerts neuroprotective effects by regulating the activation of GSK-3 and Nrf2 in a mouse model of tauopathy (20). Since the activation of GSK-3β is suppressed by phosphorylation at Ser9 by Ser/Thr protein kinases, previous researches have revealed that the Nrf2 signaling pathway is activated via AKT activation and GSK-3 inactivation (21, 22). Taken together, these findings indicate that activation of the AKT/GSK3β-Nrf2 signaling axis may contribute to the inhibition of neuroinflammation for the prevention of PD.

The researchers reveal that there is evidence that the incidence of idiopathic PD among chronic anti-inflammatory drug users is relatively low (23, 24). Considering the connection neuroinflammation with PD, dugs or ingredients with anti-inflammatory activity are highly appreciated (25). Moreover, several studies have reported that natural products have neuroprotective effect on the prevention and treatment of PD (10, 26, 27). Polydatin (3,4′,5-trihydroxystilbene-3-β-D-glucoside; PLD), a natural resveratrol glucoside, is extracted from the roots of Polygonum cuspidatum and found in cocoa-containing products, red wine, and peanuts, among other foods (28). Previous researches have revealed that PLD exhibits various biological effects, such as anti-inflammatory activity and antioxidant activity (29, 30). Moreover, PLD has been reported to cross the blood-brain barrier and prevent motor function degeneration in multiple animal models of PD (31). However, no studies have investigated whether PLD could prevent or relieve the pathogenic process of PD by inhibiting microglial activation. In our research, we explored the neuroprotective properties of PLD in an LPS-induced rat model of PD, as well as the potential anti-inflammatory mechanisms underlying the effects of PLD.

## Materials and methods

### Reagents and chemicals

LPS (*E. coli*: serotype O55:B5), apomorphine, DAPI, PLD (>95% purity), and Brusatol (BT, an Nrf2 inhibitor) were obtained from Sigma-Aldrich (St. Louis, MO, USA). MK2206 (an AKT inhibitor) and NP-12 (Tideglusib, a GSK-3β inhibitor) were purchased from Selleck, 0.25% trypsin were obtained from Invitrogen (Carlsbad, CA, USA). Penicillin-streptomycin (PS) solutions and phosphate buffered saline (PBS) were provided by (Beyotime Inst. Biotech, Beijing, China). The Fetal bovine serum (FBS) and Dulbecco's Modified Eagle's Medium (DMEM) were provided by Gibco (Grand Island, NY, USA). Rat or mouse PGE2, IL-6, IL-1β, and TNF-α ELISA kits were obtained from Biolegend (San Diego, CA, USA).

### Animal models

Nine- to eleven-week-old male Wistar rats (Baiqiuen Medical College of Jilin University, Changchun, China) were acclimated in in microisolator cages under at 22°C with a 12/12-h light/dark cycle and were provided *ad libitum* access to food and water. All rats were kept under these conditions for 2 weeks before each research. All rats were randomly allocated to the following five groups (*n* = 18 in each group): sham group, LPS group, and PLD (25, 50, or 100 mg/kg) + LPS groups. Rats were treated and injected to be LPS-induced PD models, the protocols were performed as previously described (11, 32). PLD [25, 50, or 100 mg/kg, suspended in a 0.5% sodium carboxymethylcellulose (CMC-Na) solution and then dissolved with 1 mL of PBS, once daily] were administered orally on the 3 days prior to operation. Rats in the sham-operated group were performed orally an equal volume of the vehicle solution. After LPS-injected, rats were given by gavage once a day for 4 weeks with PLD. After rats were determined by the last behavioral test, the SN of rats was separated to investigate the effect of PLD on dopaminergic neurons and microglial activation by immunohistochemical analysis and western blotting. Dopaminergic neurons were labeled with tyrosine hydroxylase (TH) (1:1,000; Abcam, Cambridge, CA, USA), and microglia were labeled with ionized calcium binding adaptor molecule-1 (IBA-1) (1:200, Proteintech, Chicago, IL, USA). The SN of remaining rats was rapidly obtained to determine the release levels of the pro-inflammatory mediators, and the protein expression levels of TH and IBA-1.

### Behavioral tests

#### Open-field test

The open-field apparatus was a 100 × 100 × 40 cm. The bottom arena of the box was carved by a 20 × 20 cm black grid. Two and four weeks after LPS was injected, rats were subjected to an open-field test to measure the effect of the PLD treatment on motor activity. Rats were tested in a quiet, low-light environment and were allowed to adapt to the environment for 5 min. PD rats were treated with an open-field test at 2 and 4 weeks after LPS injection to investigate the effect of PLD treatment on motor activity. The bottom arena of the box was washed with a 5% water-ethanol solution prior to open-field testing to avoid the effect of previous rats.

#### Rotarod motor function test

Accelerating rotation tests are commonly performed to measure the coordination and motor balance of rats with PD (33). Two and four weeks after LPS was treated, rats were performed to a rotational test to assess the effect of PLD treatment on rats' motor dysfunction. It has been reported that the apomorphine-induced rotational test is a classical and comom method to investigate the damage of the dopaminergic system and assess the behavioral dysfunction in PD model rats (34). PD rats were put onto the cylinder for a training session (10 rpm for 10 min) to adapt this test. Injected 0.5 mg/kg apomorphine, rats were putted into the cylinder for 30 min to measure the functional motor activity. The number of turns was recorded throughout the test.

### Cell culture and treatment

A murine microglia cell line, BV-2 cells, was purchased from the Cell Culture Center in Chinese Academy of Medical Sciences (Beijing, China). BV-2 cells were grown in DMEM supplemented with 10% FBS in 5% CO_2_ at 37°C relative humidity and passaged by trypsin digestion (0.05%). The medium was changed to serum-free DMEM at least 6 h prior to the treatment of PLD or LPS. BV-2 cells were pretreated with various concentrations of PLD (dissolved in PBS) for 1 h and then stimulated with LPS (100 ng/mL) for specific time.

### No assay

Nitrite, a soluble oxidation product of NO, was measured with the Griess reagent in accordance with the manufacturer's instructions. Firstly, BV-2 cells (6 × 10^5^ cells/mL) were seeded in 24-well plates and grown overnight. After BV-2 cells were treated with PLD for 1 h and stimulated with LPS for 24 h, the supernatant was collected and mixed with Griess reagent (part 1 and part 2) in a 96-well plate. The absorbance values were obtained after incubated in the dark for 20 min using a microplate reader (Synergy HT, BioTek, USA). The nitrite concentrations were calculated by the standard curve for sodium nitrite.

### Immunofluorescence assay

Immunocytochemistry-immunofluorescence (ICC-IF) experiments were performed to detect the nuclear translocation of Nrf2 and the NF-κB p65 subunit. BV-2 cells were seeded onto poly-L-lysine-coated slips in 24-well plates and cultured overnight, following which they were treated with PLD (400 μM) or LPS (100 ng/mL). The nuclear translocation of Nrf2 and the NF-κB p65 subunit were analyzed with anti-Nrf2 (1:50 Abcam, Cambridge, UK, USA) and anti-NF-κB p65 antibodies (1:100 Cell Signaling Technology, MA, USA), the procedure as previously described (11). Representative images were showed from 9 fields-of-view per treatment group.

### Immunohistochemical staining

Dopaminergic neurons in rats' SN were determined with rabbit anti-TH polyclonal antibody (1:1,000; Abcam, Cambridge, CA, USA). Microglial activation in rats' SN were detected with rabbit anti-IBA-1 polyclonal antibody (1:200, Proteintech, Chicago, IL, USA), the immunohistochemistry procedure as previously described (35). The numbers and ratios of IBA-1 and TH-positive cells were counted and then analyzed by three researchers not involved in the experimental treatments.

### Western blot analysis

The SN of PD rats and BV-2 cells were obtained for Western blot assay, the protocols as described previously (32). In this study, the membranes were incubated with primary antibodies against IBA-1 (1:1,000), TH (1:1,000), iNOS (1:1,000), COX-2 (1:1,000), Nrf2 (1:1,000) (Abcam, Cambridge, UK), AKT (1:1,000), GSK-3β (1:1,000), NF-κB p65 (1:10,000), phospho-AKT (1:1,000), phospho-GSK-3β^Ser9^ (1:1,000), phospho-NF-κB p65 (1:1,000) (Cell Signaling Technology, MA, USA), β-actin (1:10,000), and PCNA (1:1,000) (Santa Cruz, CA, USA); secondary antibodies against goat anti-rabbit (1:2,000) or goat anti-mouse (1:2,000). Next, the blots were determined with ECL Western blot detection reagents (Amersham Pharmacia Biotech), and performed in accordance with standard protocols (36).

### ELISA experiments

The rat SN was quickly dissected, placed in ice-cold PBS and then washed with ice-cold PBS. After dried and weighed, the SN of PD rats was transferred into liquid nitrogen. The SN was grinded and then diluted by PBS at a tissue weight/volume ratio of 1:5. After disrupted using an ultrasonic homogenizer, the SN tissue was centrifuged at 12,000 rpm for 20 min at 4°C. The supernatant was analyzed on the basis of manufacturer protocols of rat ELISA kits.

BV-2 cells were performed to method of 0.05 % trypsinization and seeded onto 24-well plates (2.5 × 10^5^ cells/well). Cells were pretreated with BT (200 nM) for 2 h or PLD (100, 200, or 400 μM) for 1 h, following which they were exposured to LPS (100 ng/mL) for 24 h. After the media were obtained and centrifuged, the release levels of PGE2, TNF-α, IL-1β, and IL-6 were assessed by manufacturer protocols of mouse ELISA kits.

### Statistical analysis

All data were collected from repeated experiments and presented as means ± SEM. To perform the statistical test, SPSS 19.0 software (IBM) was used. The differences between experimental groups were analyzed with one-way ANOVA, while multiple comparisons were performed with the least significant difference (LSD) method. Differences were set statistically significant at *p* < 0.05 or *p* < 0.01.

## Results

### PLD treatment ameliorates behavioral dysfunction in rats with LPS-induced PD

**Figure 1:**
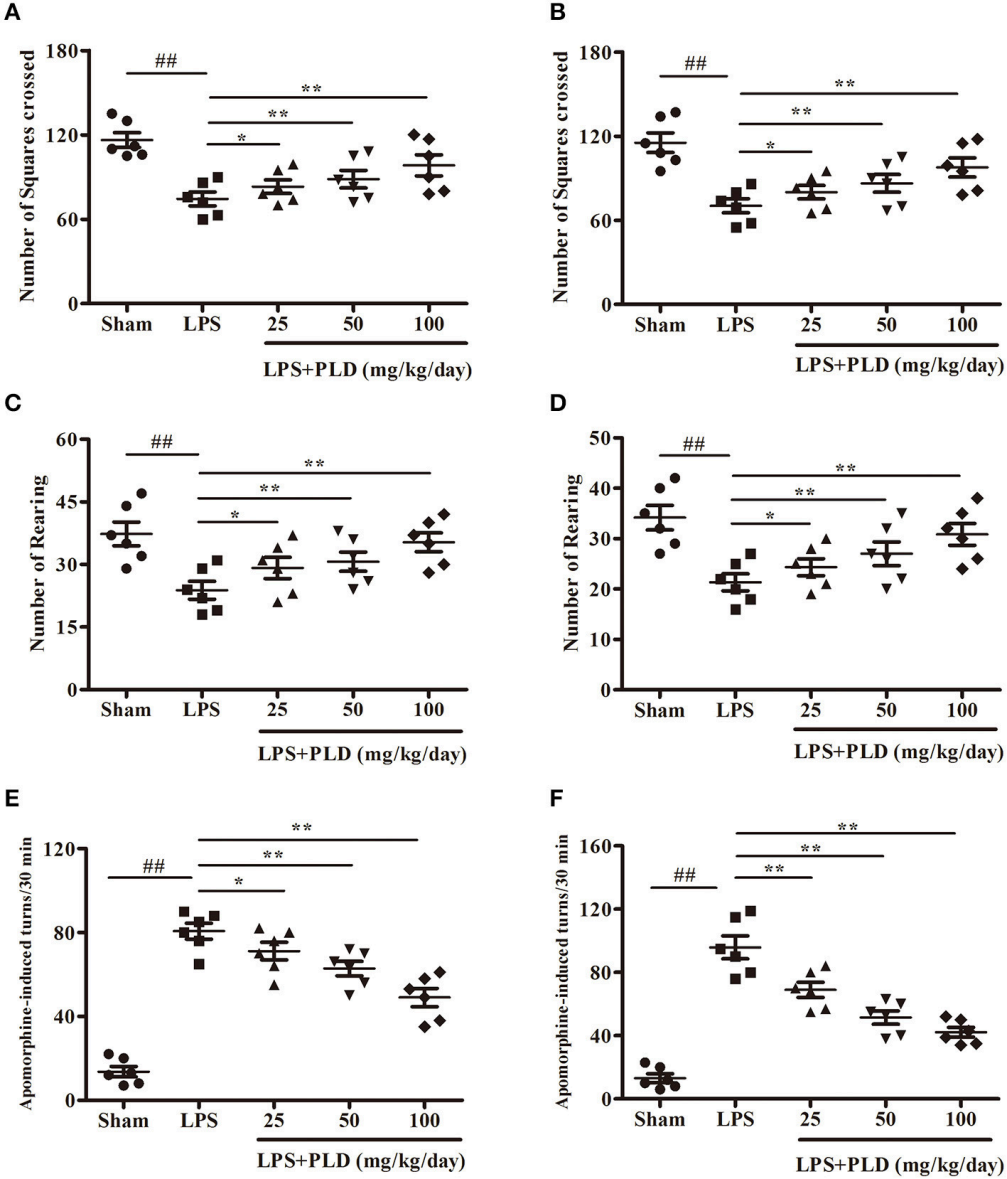
PLD treatment improves the behavioral dysfunction in rats with LPS-induced PD. Two and four weeks after LPS injection, the motor dysfunction was assessed by two behavioral tests. **(A,B)** Two and four weeks after LPS injection, the number of squares crossed in the open-field test, respectively. **(C,D)** Two and four weeks after LPS injection, the number of rearing as recorded in the open-field test, respectively. **(E,F)** Two and four weeks after LPS injection, the number of turns induced by apomorphine for LPS-induced PD model rats, respectively. All data are presented as means ± SEM (*n* = 6 in each group). ^*##*^*p* < 0.01 vs. Sham-operated control group; *p < 0.05 and **p < 0.01 vs. LPS group.

To explore the effect of PLD treatment on motor dysfunction of LPS-induced PD rats, rats were performed to behavioral tests at 2 and 4 weeks after LPS injection. The open-field test is a classic and common behavioral test used to comprehensively evaluate spontaneous behavior in PD rats. PLD treatment prominently ameliorated impairments in locomotor activity as determined by the number of squares crossed (Figures 1A,B) and rearing behavior (Figures 1C,D) during the 5-min open-field test. Considering that apomorphine treatment affects dopaminergic system and causes rotational behavior toward the side of damage, apomorphine-induced rotation is common used to assess the effect of drug on the dopaminergic system. Our results indicated that PLD treatment visibly decreased the number of apomorphine-induced rotations (Figures 1E,F). These findings suggest that PLD treatment exerted beneficial effects on motor dysfunction in rats with LPS-induced PD.

### PLD treatment increases the survival rate of dopaminergic neurons in the SN

**Figure 2:**
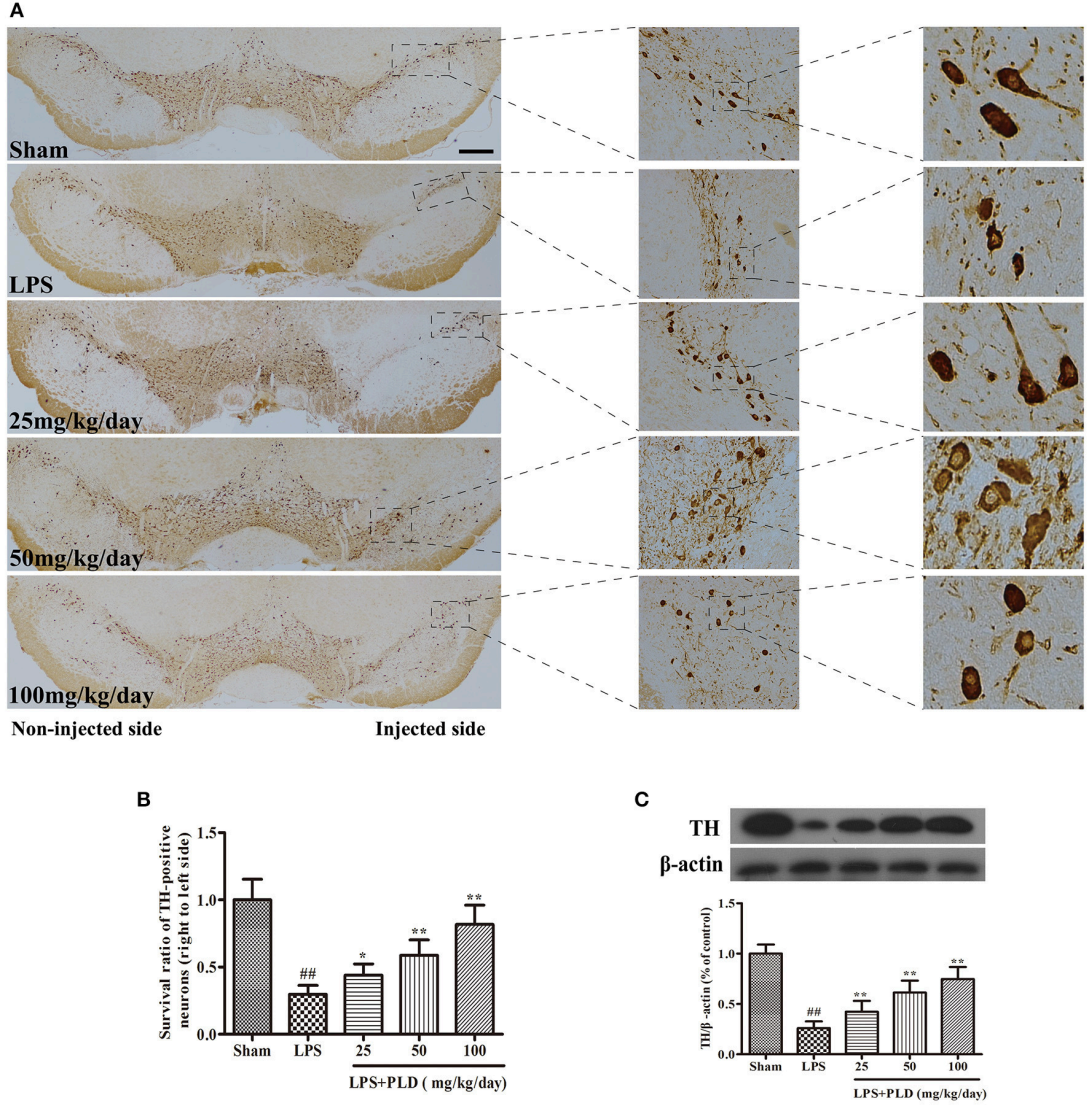
PLD treatment increases the survival rate of dopaminergic neurons in the SN. PBS or 2 μg LPS was unilaterally injected into the right SN to induce a rat model of PD. Rats were sacrificed 4 weeks after LPS injection. **(A)** Images of immunohistochemical staining for tyrosine hydroxylase (TH)-positive cells (*n* = 6 in each group); the scale bar represents 1.0 mm. **(B)** The survival ratio of dopaminergic neurons in the SN (TH-positive cells on the injected vs. non-injected side). **(C)** TH expression in the SN was determined via Western blotting (*n* = 6 in each group). β-actin was utilized as an internal control. A representative immunoblot of three independent experiments is shown. Values are presented as the mean ± SEM. ^*##*^*p* < 0.01, vs. sham-operated control group; *p < 0.05, **p < 0.01 vs. LPS group.

It has been reported that unilateral intranigral injection of LPS is common used to research the damage effects of neuroinflammation on the dopaminergic system. TH is the rate-limiting enzyme involved in the synthesis of catecholamines and participates in dopamine synthesis. In PD animal experiments, PBS or 10 μg of LPS was injected into the right SN of rats to establish animal models. Rats were sacrificed 4 weeks after LPS injection to obtain the SN. Immunohistological staining for TH in SN was conducted to further assess the protective effect of PLD on dopaminergic neurons in PD rats. We found that the number of TH-positive neurons was significantly lower in LPS-injected rats than in sham-operated rats. However, results showed that PLD treatment (25, 50, and 100 mg/kg/day) markedly suppressed the death of TH-positive neurons (Figures 2A,B). Meanwhile, we also investigated the effect of PLD on TH protein levels in the SN of rats using western blot analysis. Results showed that PLD treatment markedly increased the levels of TH protein in SN of rats (Figure 2C). Taken together, these findings indicate that PLD treatment attenuated LPS-induced damage to TH-positive neurons, suggesting that PLD increases the survival of dopaminergic neurons in PD animal model.

### PLD treatment alleviates the LPS-induced activation of microglia in the SN

**Figure 3:**
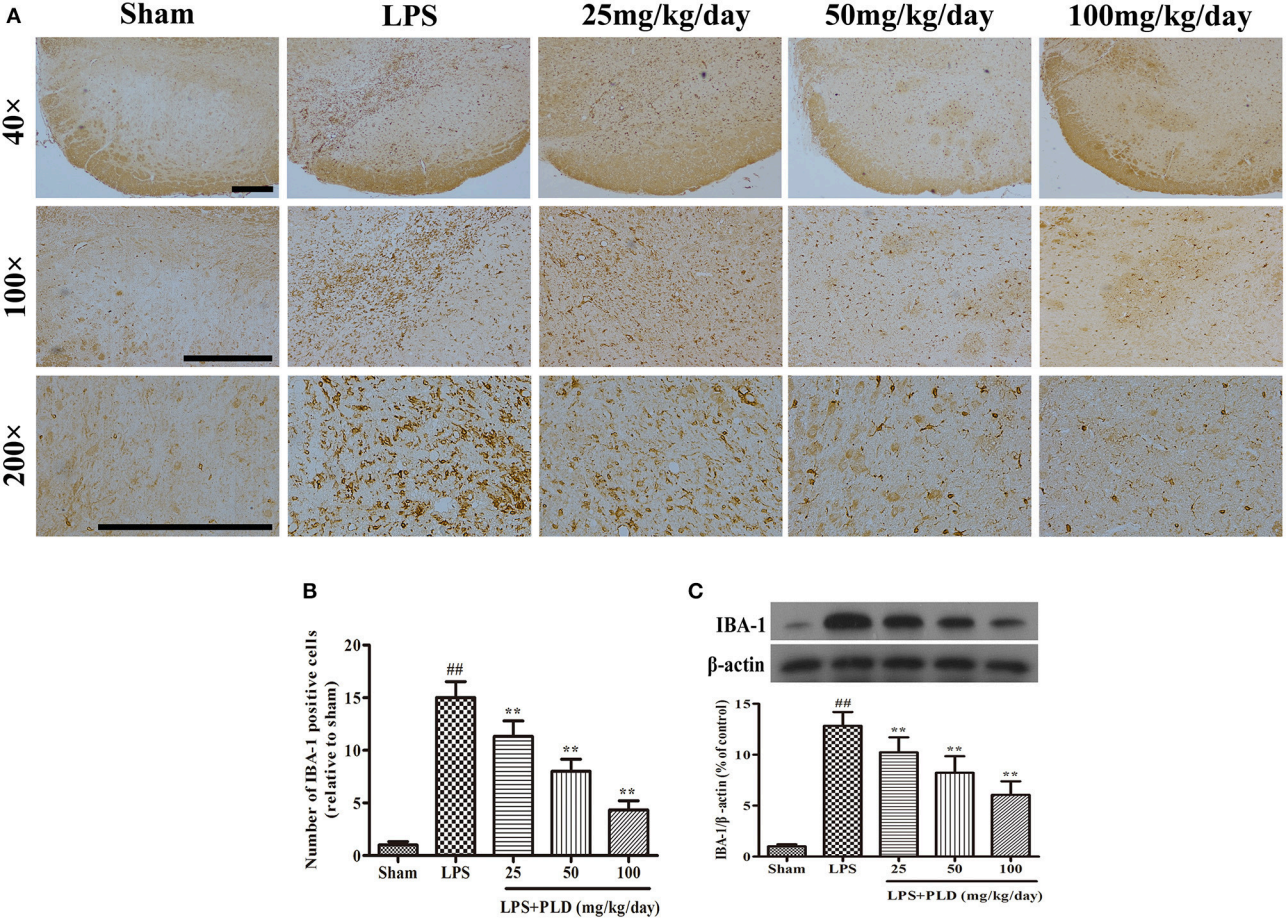
PLD treatment alleviates the LPS-induced activation of microglia in the SN. **(A)** The morphological changes of the microglia in the SN were shown via IBA-1 immunohistochemical staining (*n* = 6 per group). Representative photomicrographs of the SN area were shown. **(B)** The number of IBA-1-positive cells was calculated. **(C)** The level of the IBA-1 protein was measured via Western blotting (*n* = 6 in each group). β-actin was utilized as an internal control. Values are mean ± SEM. ^*##*^*p* < 0.01, vs. sham-operated control group; **p < 0.01 vs. LPS group.

In order to determine whether the protective effect of PLD on dopaminergic neurons is associated with an anti-neuroinflammatory response, we investigated the effect of PLD on microglial activation in LPS-induced PD rat. From 3 days prior to LPS injection to 28 days post-LPS injection, PLD was implemented orally at doses of 25, 50, and 100 mg/kg once per day. Four weeks after LPS injection, immunohistochemical and Western blot assays were performed to examine the effect of PLD on microglial activation. It has been revealed that the expression level of IBA-1 protein is a specific marker of microglial activation. We found that immunohistochemical staining experiments revealed that PLD treatment significantly inhibited microglial activation in a dose-dependent manner (Figures 3A,B). And our results indicated that PLD treatment also reduced levels of IBA-1 protein in a concentration-dependent manner (Figure 3C). Overall, these findings indicate that PLD treatment remarkably suppresses microglial activation in the SN of LPS-induced PD rats.

### PLD treatment suppresses the production of pro-inflammatory mediators in the SN

**Figure 4:**
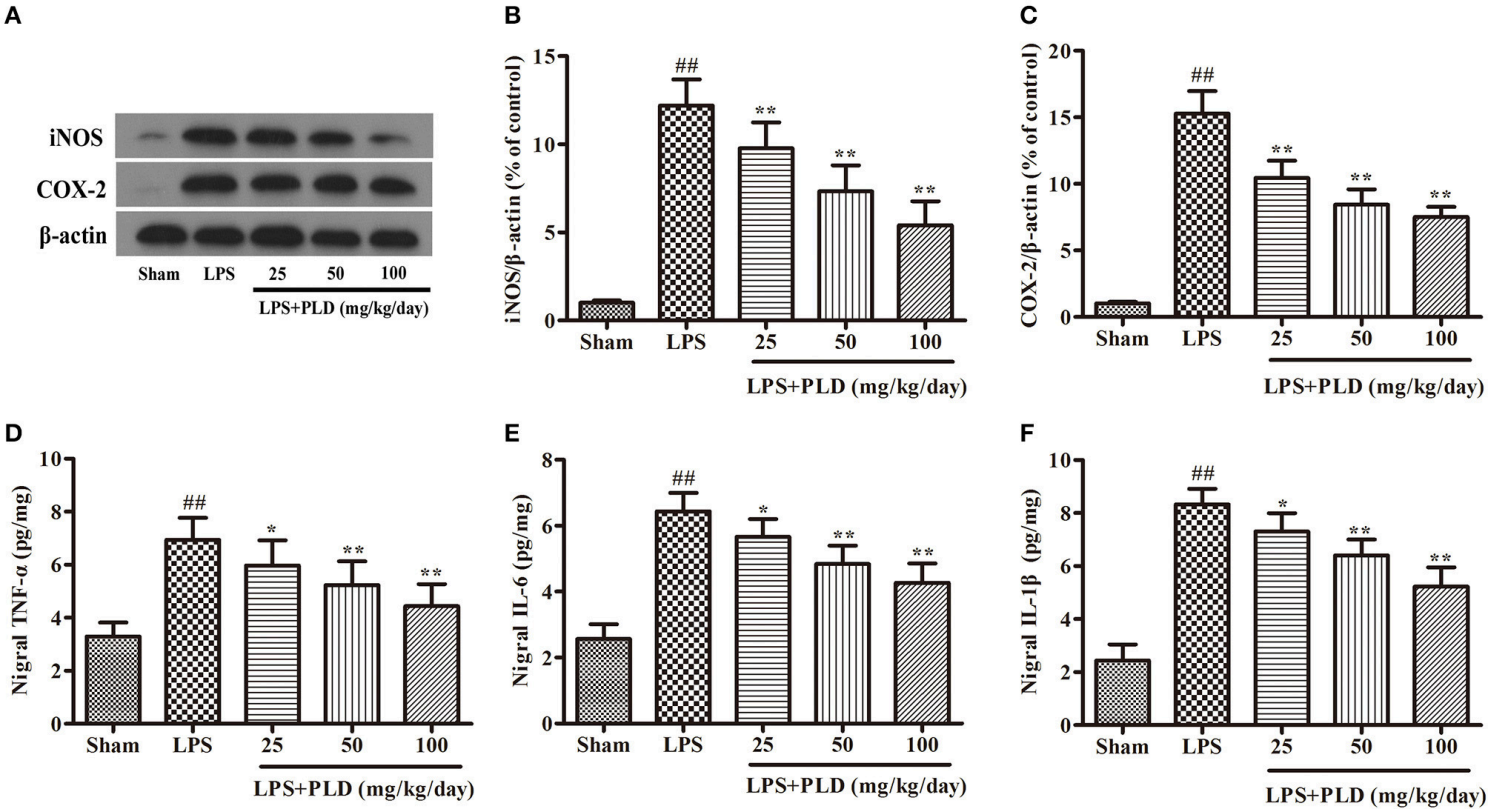
PLD treatment suppresses the production of pro-inflammatory mediators in the SN. Four weeks after LPS injection, the SN was isolated from the brains of PD rats to measure the production of pro-inflammatory mediators (iNOS, COX-2, TNF-α, IL-1β, and IL-6). **(A)** The protein levels of iNOS **(B)** and COX-2 **(C)** were measured via Western blotting. β-actin was utilized as an internal control. The release levels of TNF-α **(D)**, IL-1β **(F)**, and IL-6 **(E)** were measured via ELISA. Values are mean ± SEM (*n* = 6 in each group). ^*##*^*p* < 0.01, vs. sham-operated control group; *p < 0.05, **p < 0.01 vs. LPS group.

To further investigate the effect of PLD on neuroinflammation in the SN, we performed Western blot and ELISA experiments to examine the production of pro-inflammatory mediators in the SN. Our results revealed that LPS injection remarkably up-regulated the production of pro-inflammatory mediators in the SN, while PLD treatment significantly suppressed the production of these pro-inflammatory mediators in a dose-dependent manner (Figure 4). These results demonstrate that PLD inhibits the production of pro-inflammatory mediators in the SN of rats with LPS-induced PD.

### PLD treatment increases the expression of p-AKT, p-GSK-3β^Ser9^, and Nrf2, and downregulates the phosphorylation of NF-κB p65 in rats with LPS-induced PD

**Figure 5:**
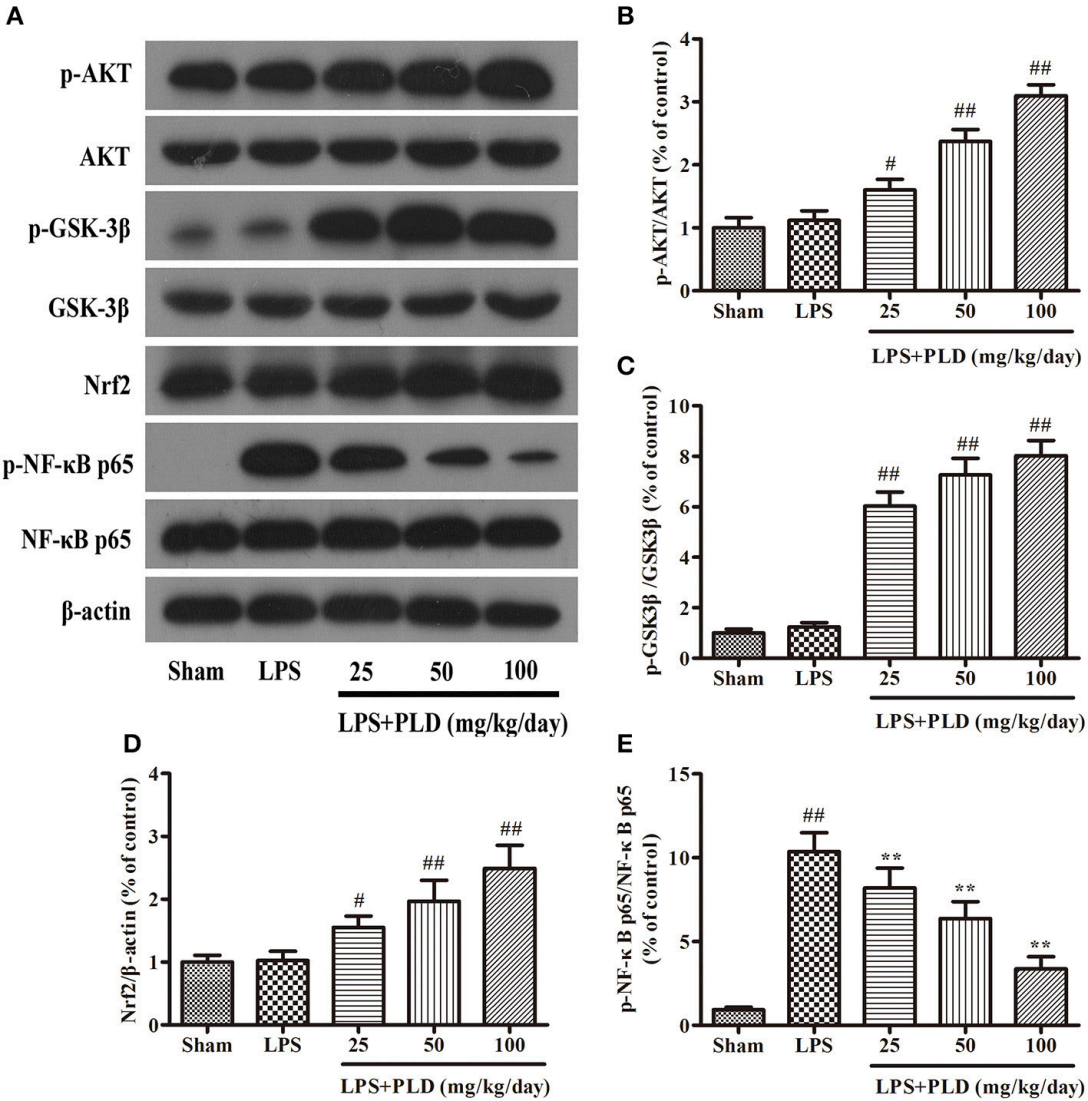
PLD treatment increases the expression of p-AKT, p-GSK-3β^Ser9^, and Nrf2, and downregulates the phosphorylation of NF-κB p65 in rats with LPS-induced PD. Four weeks after LPS injection, we measured levels of p-AKT **(A)**, p-GSK-3β^Ser9^ **(B)**, Nrf2 **(C)**, and p-NF-κB p65 **(D,E)** expression in the SN of PD rats via Western blotting. Similar results were obtained from three independent experiments. β-actin was utilized as an internal control. Values are presented as the mean ± SEM (*n* = 6 in each group). ^#^*p* < 0.05, ^*##*^*p* < 0.01 vs. sham-operated control group; **p < 0.01 vs. LPS group.

To explore the anti-inflammatory mechanisms underlying the effects of PLD in an LPS-induced model of PD, we performed Western blot analyses for protein levels of p-AKT, p-GSK-3β^Ser9^, Nrf2, and p-NF-κB p65. Previous studies have reported that pharmacological targeting of GSK-3 and Nrf2 exerts neuroprotective effects (20), and that AKT, GSK-3β, and Nrf2 activity is responsible for improvements in PD symptoms. Our results indicated that PLD treatment increased protein levels of p-AKT, p-GSK-3β^Ser9^, and Nrf2, and downregulated the phosphorylation of NF-κB p65 in rats with LPS-induced PD. These findings suggest that the anti-inflammatory effects of PLD in LPS-induced PD may be associated with increases in protein levels of p-AKT, p-GSK3β^Ser9^, and Nrf2, as well as decreases in the phosphorylation of NF-κB p65 (Figure 5).

### PLD attenuates the inflammatory response in LPS-activated BV-2 cells

**Figure 6:**
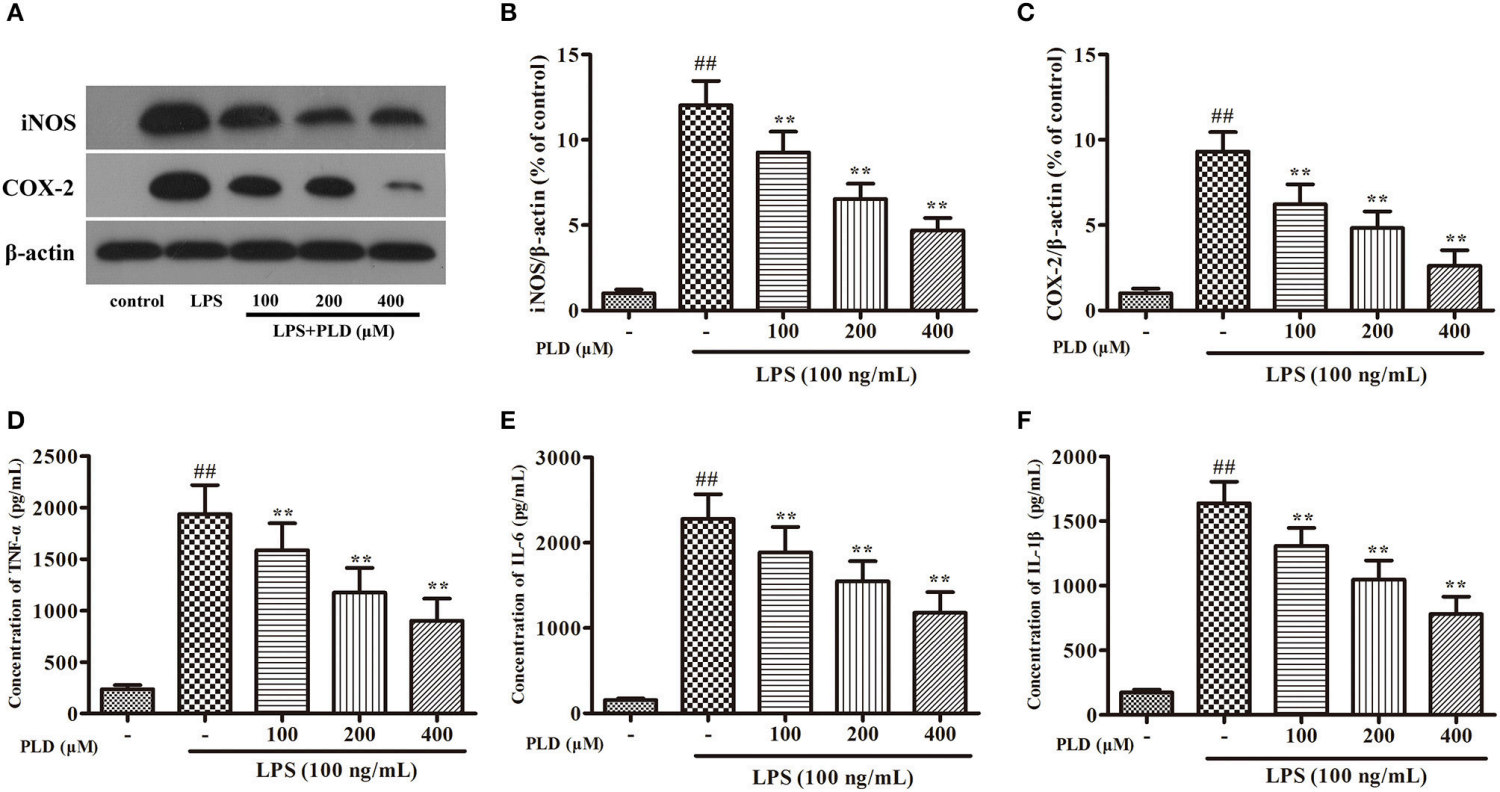
PLD attenuates the inflammatory responses in LPS-activated BV-2 cells. BV-2 cells were pretreated with various concentrations of PLD (100, 200, and 400 μM) for 1 h, and then stimulated with LPS (100 ng/mL) for another 24 h. **(A)** After BV-2 cells were collected, the protein expression of iNOS **(B)** and COX-2 **(C)** were measured via Western blotting. Levels of TNF-α **(D)**, IL-6 **(E)**, and IL-1β **(F)** in culture supernatants were measured by ELISA. Similar results were obtained from three independent experiments. Values are mean ± SEM (*n* = 4 in each group). ^*##*^*p* < 0.01, vs. control group; **p < 0.01 vs. LPS group.

Similar to primary microglia, the murine microglial BV-2 cell line produces NO, PGE2, and various cytokines following LPS stimulation. To validate whether PLD regulates microglia-mediated neuroinflammation, we first investigated the effect of PLD on the inflammatory response in BV-2 cells. We have found that less than 500 μM of PLD has no cytotoxicity on BV-2 (Figure S2). Our findings indicated that PLD suppressed the production of proinflammatory mediators (iNOS, COX-2, TNF-α, IL-1β, and IL-6) in LPS-activated BV-2 cells in a concentration-dependent manner (Figure 6). These results indicate that PLD may inhibit the inflammatory response in LPS-activated BV-2 cells.

### PLD enhances the intracellular localization of Nrf2 and inhibits the intracellular localization of the NF-κB p65 subunit in LPS-activated BV-2 cells

**Figure 7:**
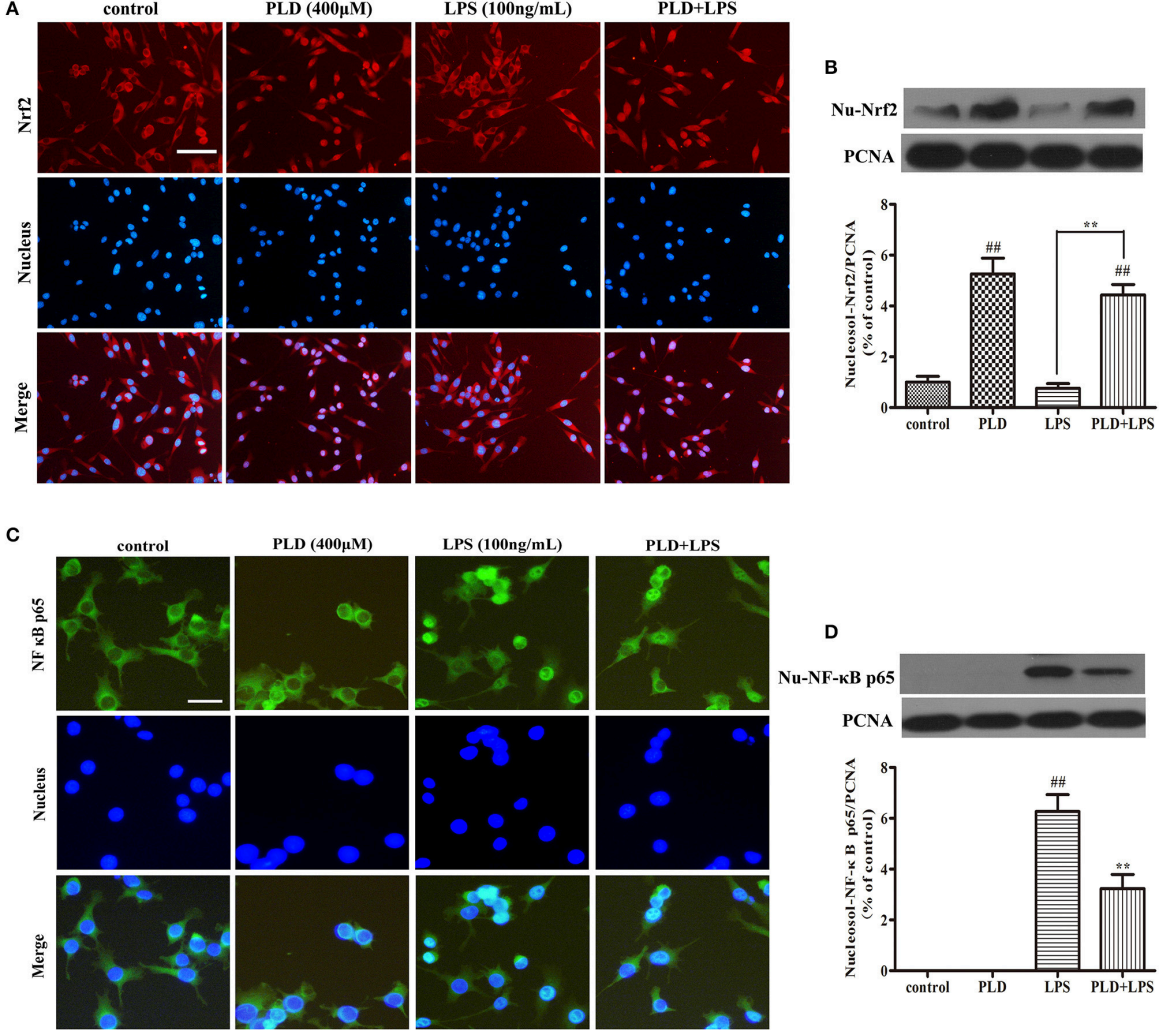
PLD enhances the nuclear translocation of Nrf2 and inhibits the nuclear translocation of the NF-κB p65 subunit in LPS-activated BV-2 cells. BV-2 cells were pretreated with PLD (400 μM) for 1 h, following which they were stimulated with LPS (100 ng/mL) for 1 h. **(A,C)** The nuclear translocation of Nrf2 and NF-κB p65 was detected via an ICC-IF assay. Representative photomicrographs of Nrf2 and NF-κB p65 expression are shown. **(B,D)** The intranuclear protein expression of Nrf2 and NF-κB p65 was measured via Western blotting. Similar results were obtained from three independent experiments. PCNA was utilized as a nucleosol internal control. Values are presented as the mean ± SEM (*n* = 4 in each group). ^*##*^*p* < 0.01, vs. control group; **p < 0.01 vs. LPS group.

In our *in vivo* model of LPS-induced PD, PLD enhanced the activation of Nrf2 and inhibited the activation of NF-κB. To further demonstrate whether the effect of PLD on neuroinflammation is associated with these two signaling pathways, BV-2 cells were pretreated with PLD (400 μM) for 1 h, followed by LPS for 1 h (100 ng/mL). Previous studies have indicated that activation of Nrf2 and NF-κB signaling pathways may cause nuclear translocation of Nrf2 (37) and the NF-κB p65 (38) subunit. Because the nuclear translocation of Nrf2 plays a key role in inhibiting the inflammatory response, we investigated whether PLD upregulates the nuclear translocation of Nrf2. Indeed, our results indicated that PLD efficiently promoted the nuclear translocation of Nrf2 (Figures 7A,B). Furthermore, LPS treatment exerted little-to-no effect on the nuclear translocation of Nrf2 in BV-2 cells (Figures 7A,B). These findings demonstrate that activation of the NF-κB signaling pathway upregulates inflammation in microglia. The activation of the NF-κB signaling pathway mainly represents the nuclear translocation of the NF-κB p65 subunit. Our results indicated that LPS treatment induced the nuclear translocation of NF-κB p65, while pretreatment with PLD dramatically suppressed LPS-induced nuclear translocation of NF-κB p65 in BV-2 cells (Figures 7C,D).

### PLD activates the AKT/GSK3β-Nrf2 signaling axis in BV-2 cells

**Figure 8:**
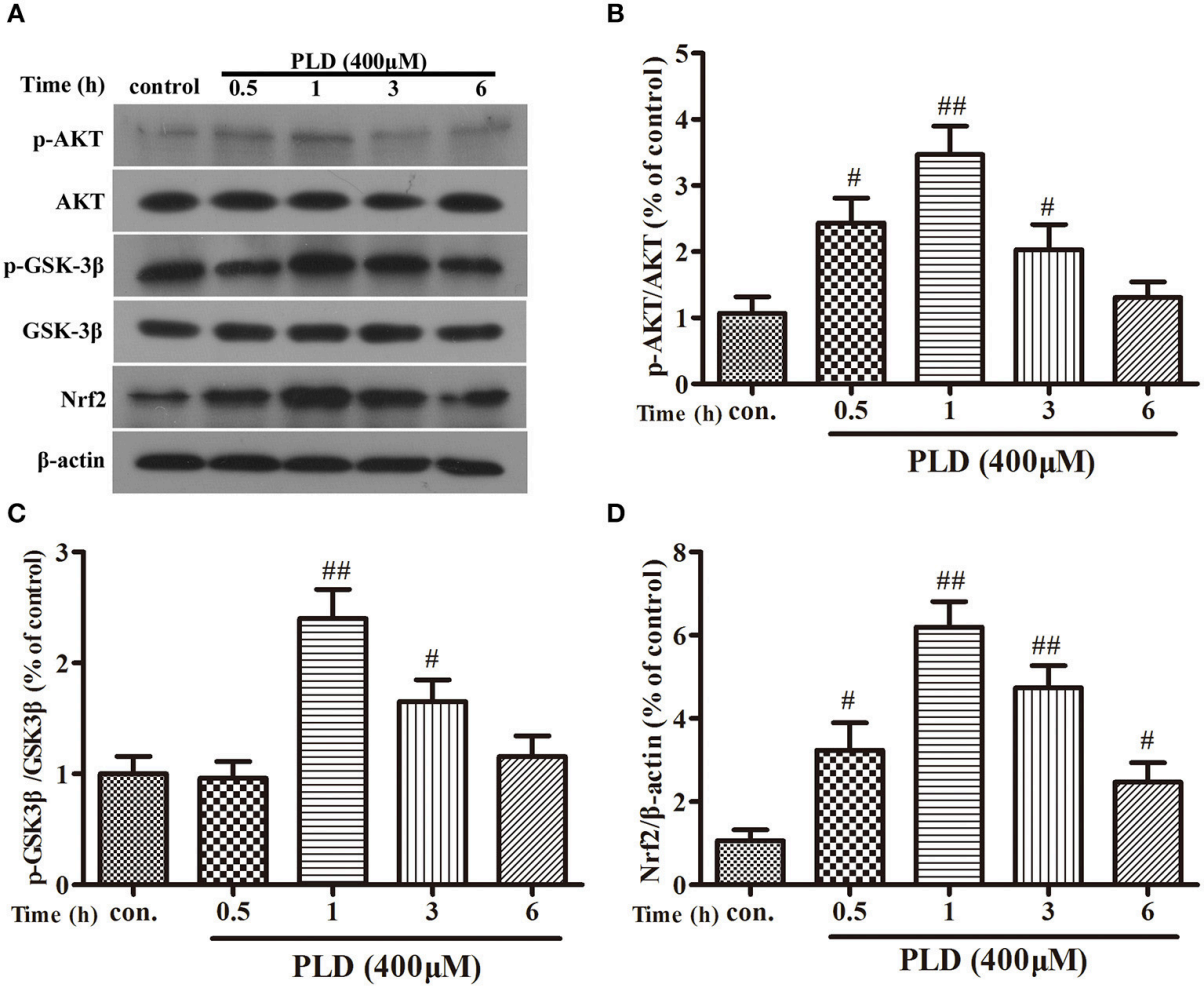
PLD upregulates the phosphorylation of AKT and GSK3β, and the expression of Nrf2 in BV-2 cells. **(A)** BV-2 cells were treated with PLD (400 μM) for different time (0, 0.5, 1, 3, 6 h). After cells were harvested, the protein expression of AKT **(B)**, p-AKT, GSK-3β, p-GSK-3β^Ser9^ **(C)**, and Nrf2 **(D)** was measured via Western blotting. β-actin was utilized as an internal control. Similar results were obtained from three independent experiments. Values are mean ± SEM (*n* = 4 in each group). ^#^*p* < 0.05, ^*##*^*p* < 0.01 vs. control group.

**Figure 9:**
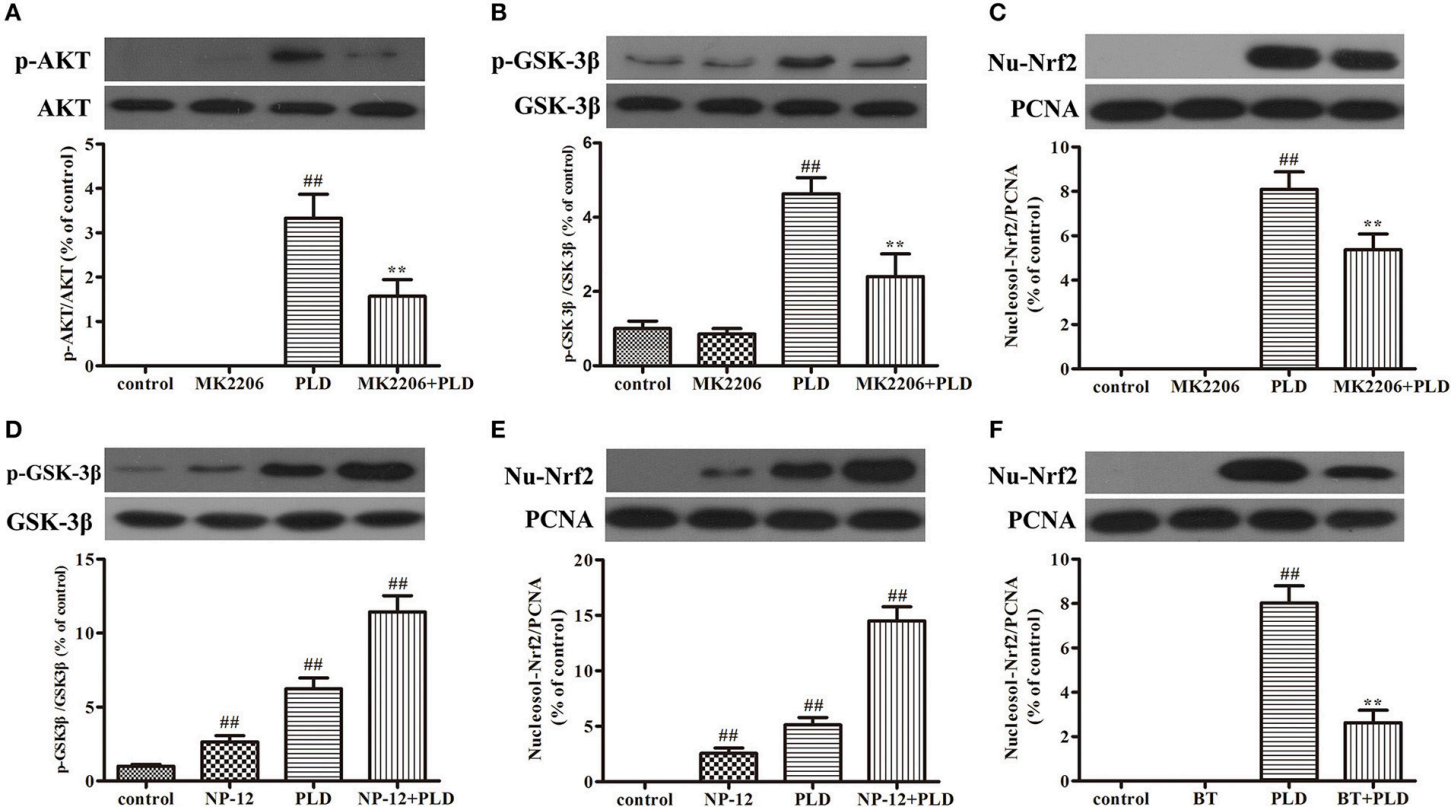
PLD activates the AKT/GSK3β-Nrf2 signaling axis in BV-2 cells. BV-2 cells were pretreated with MK2206 (an inhibitor of AKT, 10 μM) for 2 h, following which they were treated with PLD for 1 h. After cells were harvested, the protein expression of AKT, p-AKT **(A)**, GSK3β, p-GSK-3β^Ser9^ **(B)**, and Nucleosol-Nrf2 **(C)** was measured via Western blotting. BV-2 cells were pretreated with NP-12 (an inhibitor of GSK-3β, 2.5 μM) for 4 h, following which they were treated with PLD for 1 h. After cells were harvested, the protein expression of GSK-3β, p-GSK-3β^Ser9^ **(D)**, and Nucleosol-Nrf2 **(E)** was measured via Western blotting. BV-2 cells were pretreated with BT (Brusatol: an inhibitor of Nrf2, 200 nM) for 6 h, following which they were treated with PLD for 1 h. After cells were harvested, the protein expression of Nucleosol-Nrf2 **(F)** was measured via Western blotting. β-actin was utilized as an internal control, while PCNA was utilized as a nucleosol internal control. Similar results were obtained from three independent experiments. Values are presented as the mean ± SEM (*n* = 4 in each group), ^*##*^*p* < 0.01 vs. control group, **p < 0.01 vs. PLD group.

PLD upregulated the AKT/GSK-3β phosphorylation levels and Nrf2 expression level in the SN in our *in vivo* model of LPS-induced PD. Several previous studies have indicated that Nrf2 expression is regulated by AKT and GSK-3β (20, 37, 39, 40). And AKT is the upstream of GSK-3β and can regulate the activation of GSK-3β. To investigate whether the effect of PLD on Nrf2 is regulated by AKT and GSK-3β, BV-2 cells were pretreated with MK2206 (an inhibitor of AKT) or NP-12 (an inhibitor of GSK3β). After microglia were treated with PLD for various durations (0, 0.5, 1, 3, and 6 h), total levels of Nrf2 protein expression were measured via Western blotting. Our results indicated that PLD treatment upregulated the total protein expression of Nrf2 in BV-2 cells. Because Nrf2 expression was remarkable in BV-2 cells treated with PLD for 1 h, we also investigated the effect of different PLD treatment durations on p-AKT and p-GSK-3β^Ser9^ expression in microglia (0, 0.5, 1, 3, and 6 h). Similarly, p-AKT and p-GSK-3β^*Ser*9^ expression was notable in BV-2 cells treated with PLD for 1 h. Consistent with our *in vivo* findings, these results indicate that PLD may increase the phosphorylation of AKT and GSK-3β to upregulate the expression of Nrf2 in BV-2 cells (Figure 8). Subsequently, we measured the effect of MK2206 on AKT/GSK-3β^Ser9^ phosphorylation and Nrf2 expression, observing that PLD-induced Nrf2 activation and GSK-3β^Ser9^ phosphorylation were effectively blocked by treatment with MK2206 (Figures 9A–C). We also measured the effect of NP-12 on the GSK-3β^Ser9^ phosphorylation and Nrf2 expression. Our results indicated that NP-12 treatment substantially increased PLD-induced GSK-3β^*Ser*9^ phosphorylation and Nrf2 activation (Figures 9D,E). Taken together, these results suggest that PLD treatment activates the AKT/GSK3β-Nrf2 signaling axis in BV-2 cells.

### PLD suppresses the phosphorylation of NF-κB p65 and the release of pro-inflammatory mediators in LPS-activated BV-2 cells via Nrf2 activation

**Figure 10:**
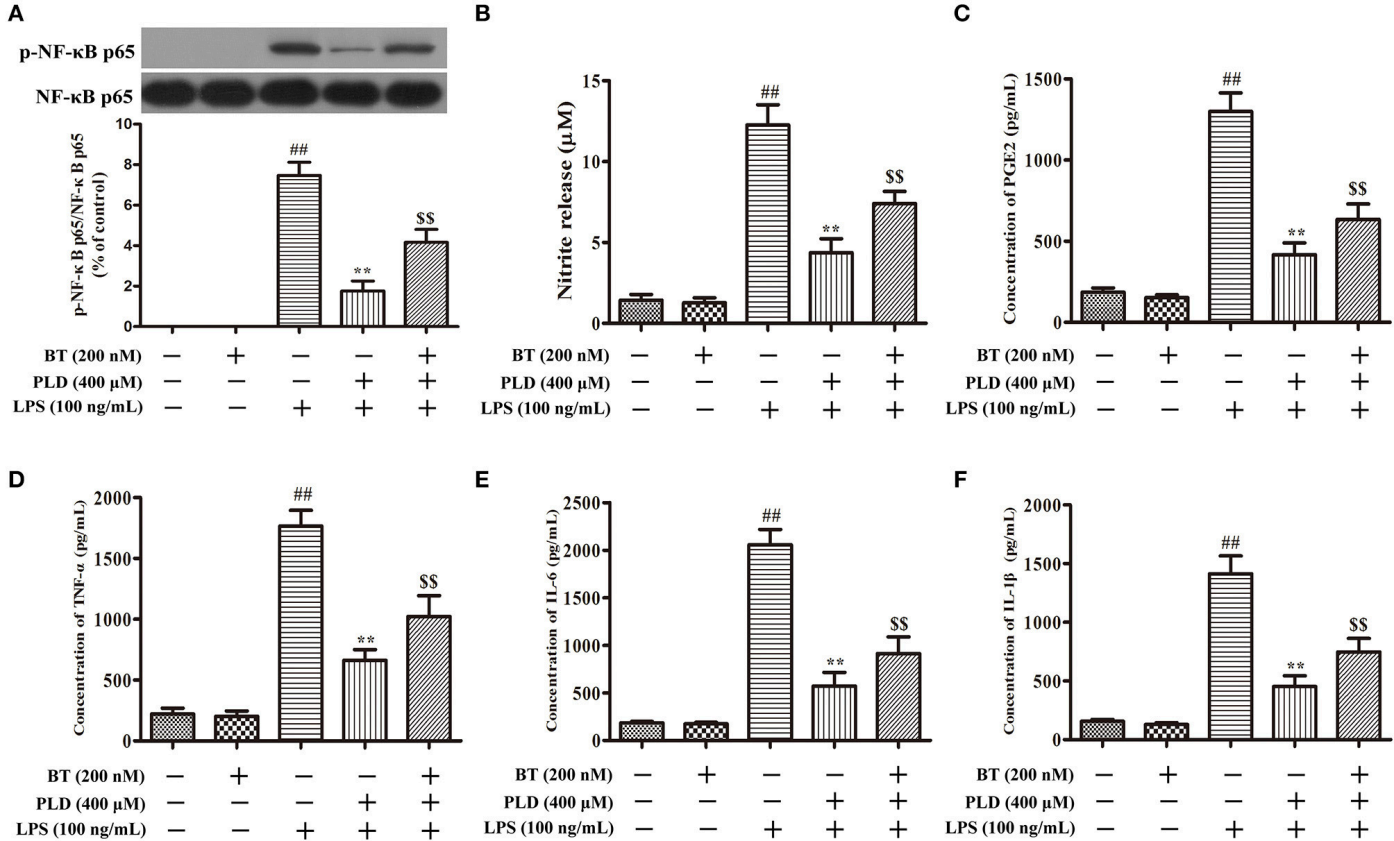
PLD suppresses the phosphorylation of NF-κB p65 and the release of pro-inflammatory mediators in LPS-activated BV-2 cells via Nrf2 activation. Following pretreatment with BT (Brusatol: an inhibitor of Nrf2, 200 nM) for 6 h, BV-2 cells were treated with PLD for 1 h and then stimulated with LPS for 1 h. **(A)** After cells were harvested, the protein expression of p-NF-κB p65 and NF-κB p65 was measured via Western blotting. **(B)** Levels of NO in culture supernatants were measured using the Griess reagent. Levels of PGE2 **(C)**, TNF-α **(D)**, IL-6 **(E)**, and IL-1β **(F)** in culture supernatants were measured via ELISA. Similar results were obtained from three independent experiments. Values are presented as the mean ± SEM (*n* = 4 in each group), ^*##*^*p* < 0.01, vs. control group; **p < 0.01, vs. LPS group; ^*$$*^*p* < 0.01, vs. BT+PLD+LPS group.

To determine whether the anti-inflammatory effect of PLD is related to Nrf2 activation, BV-2 cells were pretreated with BT (an inhibitor of Nrf2) (Figure 9F). Our findings indicated that BT pretreatment attenuated the inhibitory effect of PLD on the phosphorylation of NF-κB p65. The release level of NO and PGE2 is regulated by iNOS and COX-2, respectively. NO and PGE2 are the important components of neuroinflammation and participate in cell survival and death in the pathological processes of neurodegenerative diseases. We found that PLD memorably suppressed the release level of neurotoxic factors (NO, PGE2, TNF-α, IL-1β, and IL-6) in LPS-treated BV-2 cells. However, BT pretreatment attenuated the inhibitory effect of PLD on the release of pro-inflammatory mediators. These results suggest that the pretreatment regimen effectively suppressed the phosphorylation of NF-κB p65 and the release of pro-inflammatory mediators in LPS-activated BV-2 cells via Nrf2 activation (Figure 10).

## Discussion

In the present study, we aimed to determine whether PLD protects against dopaminergic neurodegeneration by inhibiting the activation of microglia. Our findings demonstrated that PLD treatment ameliorates behavioral dysfunction in rats with LPS-induced PD. Furthermore, our results indicated that PLD treatment prevented the loss of dopaminergic neurons in the SN. While LPS administration significantly upregulated the expression of IBA-1 and proinflammatory mediators in the SN, treatment with PLD significantly attenuated these effects, suggesting that PLD suppresses neuroinflammation due to overaction of microglia in a rat model of PD. And we have found that PLD suppressed M1 microglia phenotype and enhanced M2 microglia phenotype in activated microglia (Figure S1). To further investigate the neuroprotective mechanisms of PLD, we measured the production of pro-inflammatory mediators as well as levels of p-AKT, p-GSK-3β^Ser9^, Nrf2, and p-NF-κB p65 expression in BV-2 cells. Our findings indicated that PLD enhanced the production of p-AKT, p-GSK-3β^Ser9^, and Nrf2 while suppressing NF-κB p65 activation in microglia. These results suggest that PLD prevented dopaminergic neurodegeneration due to microglial activation via activation of the AKT/GSK3β-Nrf2 signaling axis.

PD is a global neurodegenerative movement disorder whose primary cause remains elusive. Nonetheless, previous studies have indicated that the inflammation and immune activation in brain contribute to the pathophysiology of PD (41). Activated microglia and astrocytes could produce reactive oxygen intermediates, NO, and inflammatory cytokines, which lead to neuroinflammatory activities resulting in neurodegeneration. Therefore, an understanding of the neuroinflammatory mechanisms and key biomolecules that control microglial activation is indispensable for developing a novel therapeutic strategy for the prevention of dopaminergic neurodegeneration in patients with PD.

In PD research, various PD models are established and used to explore the pathogenesis of PD. For instance, 6-hydroxydopamine (6-OHDA) is used to establish a PD model through oxidative stress, 1-methyl-4-phenyl-1,2,3,6-tetrahydropyridine (MPTP) and rotenone through mitochondrial complex I inhibition, and LPS is used to establish a PD model through its glial cell activation. It has been reported that unilateral stereotaxic injection of LPS into the rat's SN leads to microglial over-activation, which selectively produces lasting degeneration of dopaminergic neurons resulting in the pathological and clinical features of PD (42). Therefore, LPS-induced PD model is performed to mimic the effect of neuroinflammation on brain.

Microglia, resident macrophages of the nervous system, represents the first line of defense against infection or injury to the nervous system (43). It has been summarized that the excessive release of these pro-inflammatory mediators causes damage of dopaminergic neurons, which is then toxic to neighboring neurons and lead to the death of neurons, representing a perpetual cycle of neuronal death (44). Therefore, the inhibition of microglia-mediated neuroinflammation presents a feasible approach for the prevention and treatment of PD. In the present study, microglia is replaced by microglial line BV-2 cells to explore the anti-neuroinflammatory effects and mechanisms of PLD. Although BV-2 cells are not a complete replacement for microglia, BV-2 cells possess many features of microglia and are usually used to research neuroinflammation induced by activated microglia.

NF-κB, a transcription factor, regulates the expression of pro-inflammatory enzymes and cytokines, which contribute to amplification of inflammation response leading to neuronal damage (45). Activation of the NF-κB signaling pathway may lead to the phosphorylation and translocation of NF-κB p65, in turn upregulating the inflammatory response, which may be associated with the pathogenesis of PD (46). Such findings suggest that the inhibition of NF-κB plays a key role in the prevention and treatment of PD. In the present study, PLD suppressed the activation of NF-κB, thereby downregulating neuroinflammatory responses in both a rat model of PD and activated microglia.

Nrf2 plays an integral role in microglia-mediated protection of neurons from inflammatory responses (47, 48). Moreover, previous studies involving Nrf2-knockout mice have demonstrated that loss of Nrf2 can exacerbate neurodegenerative phenotypes (49–51). Additional studies have revealed that activation of Nrf2 downregulates neuroinflammatory responses in microglia (52). A previous study has demonstrated that 100 mg/kg/d of PLD treatment alone have little or no effect on animal studies (31). In the present study, PLD upregulated the expression of Nrf2 in a rat model of PD and in activated microglia. To evaluate the potential link between the neuroprotective effects of PLD and Nrf2 activation, we investigated whether pretreatment with the Nrf2 inhibitor BT attenuates the inhibitory effect of PLD on the release of proinflammatory mediators in activated microglia. As detailed in Figure 10, our findings indicated that PLD treatment inhibited the production of proinflammatory mediators via the activation of Nrf2.

Next, we further explored the mechanisms involved in PLD-induced Nrf2 activation. Previous studies have reported that the effect of GSK-3β on the inflammatory response is associated with the expression and nuclear localization of Nrf2 (37, 53). AKT activation is necessary for the phosphorylation of GSK-3β^Ser9^, which serves as a potential modulator of the inflammatory response (39, 40). Moreover, several studies have demonstrated that Nrf2 may be upregulated via activation of AKT and permanent inactivation of GSK-3 (54–56). Therefore, in the present study, BV-2 cells were pretreated with inhibitors of AKT, GSK-3β, and Nrf2 to determine the effect of PLD on AKT/GSK-3β^Ser9^ phosphorylation and Nrf2 expression. Our findings indicated that PLD treatment increased the phosphorylation of AKT and GSK-3β^Ser9^, as well as the expression of Nrf2, in BV-2 cells (Figure 8). Moreover, BV-2 cells were pretreated with the AKT inhibitor MK2206 in order to determine whether the expression of p-GSK-3β^Ser9^ and Nrf2 is induced via AKT activation in PLD-treated microglia. Our results indicated that PLD upregulated the phosphorylation of GSK-3β^Ser9^ and the intracellular protein expression of Nrf2 via the activation of AKT (Figures 9A–C). To investigate whether the intracellular protein expression of Nrf2 is regulated by GSK-3β inactivation, BV-2 cells were pretreated with the GSK-3β inhibitor NP-12. Our results demonstrated that PLD upregulated the intranuclear level of Nrf2 via the inactivation of GSK-3β (Figures 9D,E), suggesting that PLD treatment exerts anti-inflammatory effects by activating the AKT/GSK3β-Nrf2 signaling axis in BV-2 cells.

In our study, we only investigate the effect of PLD on murine microglial line BV-2 cells. Astrocytes are also the most abundant glial cells in the mammalian brain and play both beneficial and detrimental roles in PD. Activated astrocyte also play important roles in neuroinflammation in the pathophysiology of PD. Maybe PLD can also inhibit the activation of astrocyte to exert neuroprotective effect in PD. Although we found that PLD inhibited microglia-mediated neuroinflammation, the precise molecular mechanisms and protein targets remain elusive. A significant challenge with quantitative chemical proteomics approaches will be determining the cellular molecular target of PLD.

## Conclusions

**Figure 11:**
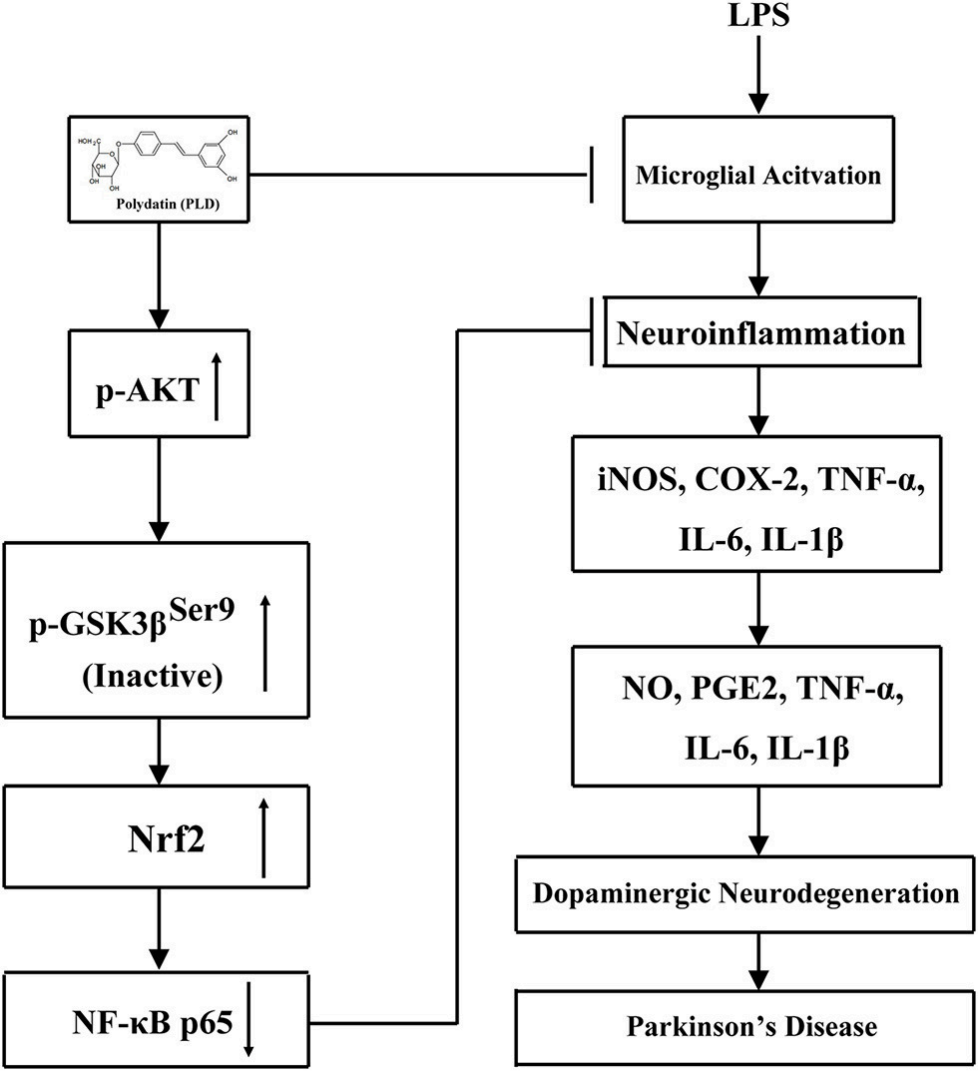
Scheme summarizing the anti-inflammatory effects of PLD on LPS-induced PD via regulation of AKT/GSK3β-Nrf2/NF-κB signal axis. Polydatin (PLD) treatment effectively prevented LPS-induced PD from microglia-mediated neuroinflammation via regulation of AKT/GSK3β-Nrf2/NF-κB signal axis.

In conclusion, the present study is the first to demonstrate that PLD effectively prevented dopaminergic neurodegeneration from inflammation-mediated damage by inhibiting the production of pro-inflammatory mediators and activation of the NF-κB signaling pathway via activation of the AKT/GSK3β-Nrf2 signaling axis (Figure 11). Taken together, these findings support the notion that PLD can be applied as a preventative treatment for PD. Considering the extensive biological activities and food source of PLD, future clinical studies are proposed to determine whether PLD can be researched and developed as a new food supplement for the prevention or intervention of PD.

## Ethics statement

This work was performed in accordance with approved animal protocols and guidelines established by the Institutional Animal Care and Use Committee of Jilin University (Changchun, China) (approved on February 27, 2015; Protocol No. 2015047). We done our best to minimize animal suffering and to reduce the number of animals used.

## Author contributions

JL and DL conceived and designed the study. BH, SF, and WW performed the experiments, analyzed the data, and wrote the paper. YL, DH, XR, TM, GC, WG, and XK collected the samples and information. All authors reviewed the manuscript. In addition, all authors have read and approved the manuscript.

### Conflict of interest statement

The authors declare that the research was conducted in the absence of any commercial or financial relationships that could be construed as a potential conflict of interest.

## Abbreviations

PD: Parkinson's disease
SN: substantia nigra
TNF-α: tumor necrosis factor alpha
IL-1β: interleukin 1β
IL-6: interleukin 6
NO: nitric oxide
PGE2: prostaglandin E2
LPS: lipopolysaccharide
Nrf2: nuclear factor-erythroid 2-related factor 2
NF-κB: nuclear factor-kappa light chain enhancer of B cells
iNOS: inducible nitric oxide synthase
GSK-3β: glycogen synthase kinase 3β
PLD: polydatin
BT: Brusatol
PS: penicillin-streptomycin
PBS: phosphate buffered saline
DMEM: Dulbecco's Modified Eagle's Medium
FBS: fetal bovine serum
SNpc: substantia nigra pars compacta
AP: anteroposterior
LAT: lateral
DV: dorsoventral
CMC-Na: sodium carboxymethylcellulose
TH: tyrosine hydroxylase
IBA-1: ionized calcium binding adaptor molecule 1
ICC-F: immunocytochemistry-immunofluorescence
PMSF: phenyl-methylsulfonyl fluoride
PVDF: polyvinylidene difluoride
6-OHDA: 6-hydroxydopamine
MPTP: 1-methyl-4-phenyl-1,2,3,6-tetrahydropyridine.

## References

[1] Brown CA Cheng EM Hays RD Vassar SD Vickrey BG. SF-36 includes less Parkinson Disease (PD)-targeted content but is more responsive to change than two PD-targeted health-related quality of life measures. Qual Life Res. (2009) 18:1219–37. 10.1007/s11136-009-9530-y 19714487 PMC2759458

[2] Blum D Torch S Lambeng N Nissou M Benabid AL Sadoul R. Molecular pathways involved in the neurotoxicity of 6-OHDA, dopamine and MPTP: contribution to the apoptotic theory in Parkinson's disease. Prog Neurobiol. (2001) 65:135–72. 10.1016/S0301-0082(01)00003-X 11403877

[3] Collier TJ Sortwell CE. Therapeutic potential of nerve growth factors in Parkinson's disease. Drugs Aging (1999) 14:261–87. 10.2165/00002512-199914040-00003 10319241

[4] Jankovic J. Parkinson's disease: clinical features and diagnosis. J Neurol Neurosurg Psychiatry (2008) 79:368–76. 10.1136/jnnp.2007.131045 18344392

[5] Gyoneva S Shapiro L Lazo C Garnier-Amblard E Smith Y Miller GW. Adenosine A2A receptor antagonism reverses inflammation-induced impairment of microglial process extension in a model of Parkinson's disease. Neurobiol Dis. (2014) 67:191–202. 10.1016/j.nbd.2014.03.004 24632419 PMC4072497

[6] Deleidi M Gasser T. The role of inflammation in sporadic and familial Parkinson's disease. Cell Mol Life Sci. (2013) 70:4259–73. 10.1007/s00018-013-1352-y 23665870 PMC11113951

[7] Sugama S Yang L Cho BP DeGiorgio LA Lorenzl S Albers DS. Age-related microglial activation in 1-methyl-4-phenyl-1,2,3,6-tetrahydropyridine (MPTP)-induced dopaminergic neurodegeneration in C57BL/6 mice. Brain Res. (2003) 964:288–94. 10.1016/S0006-8993(02)04085-4 12576189

[8] McGeer PL McGeer EG. Inflammation and neurodegeneration in Parkinson's disease. Parkinsonism Relat Disord. (2004) 10 (Suppl. 1):S3–7. 10.1016/j.parkreldis.2004.01.005 15109580

[9] Gao HM Zhou H Zhang F Wilson BC Kam W Hong JS. HMGB1 acts on microglia Mac1 to mediate chronic neuroinflammation that drives progressive neurodegeneration. J Neurosci. (2011) 31:1081–92. 10.1523/JNEUROSCI.3732-10.2011 21248133 PMC3046932

[10] Wang X Wang C Wang J Zhao S Zhang K Wang J. Pseudoginsenoside-F11 (PF11) exerts anti-neuroinflammatory effects on LPS-activated microglial cells by inhibiting TLR4-mediated TAK1/IKK/NF-kappaB, MAPKs and Akt signaling pathways. Neuropharmacology (2014) 79:642–56. 10.1016/j.neuropharm.2014.01.022 24467851

[11] Huang BX Liu JX Ju C Yang DX Chen GX Xu SY. Licochalcone a prevents the loss of dopaminergic neurons by inhibiting microglial activation in lipopolysaccharide (LPS)-induced Parkinson's disease models. Int J Mol Sci. (2017) 18:E2043. 10.3390/ijms18102043 28937602 PMC5666725

[12] Castano A Herrera AJ Cano J Machado A. Lipopolysaccharide intranigral injection induces inflammatory reaction and damage in nigrostriatal dopaminergic system. J Neurochem. (1998) 70:1584–92. 10.1046/j.1471-4159.1998.70041584.x 9580157

[13] Herrera AJ Castano A Venero JL Cano J Machado A. The single intranigral injection of LPS as a new model for studying the selective effects of inflammatory reactions on dopaminergic system. Neurobiol Dis. (2000) 7:429–47. 10.1006/nbdi.2000.0289 10964613

[14] Singh S Vrishni S Singh BK Rahman I Kakkar P. Nrf2-ARE stress response mechanism: a control point in oxidative stress-mediated dysfunctions and chronic inflammatory diseases. Free Radic Res. (2010) 44:1267–88. 10.3109/10715762.2010.507670 20815789

[15] Negi G Kumar A Sharma SS. Nrf2 and NF-kappaB modulation by sulforaphane counteracts multiple manifestations of diabetic neuropathy in rats and high glucose-induced changes. Curr Neurovasc Res. (2011) 8:294–304. 10.2174/156720211798120972 22023613

[16] Negi G Kumar A Sharma SS. Melatonin modulates neuroinflammation and oxidative stress in experimental diabetic neuropathy: effects on NF-kappaB and Nrf2 cascades. J Pineal Res. (2011) 50:124–31. 10.1111/j.1600-079X.2010.00821.x 21062351

[17] Li W Khor TO Xu C Shen G Jeong WS Yu S. Activation of Nrf2-antioxidant signaling attenuates NFkappaB-inflammatory response and elicits apoptosis. Biochem Pharmacol. (2008) 76:1485–9. 10.1016/j.bcp.2008.07.017 18694732 PMC2610259

[18] Ganesh Yerra V Negi G Sharma SS Kumar A. Potential therapeutic effects of the simultaneous targeting of the Nrf2 and NF-kappaB pathways in diabetic neuropathy. Redox Biol. (2013) 1:394–7. 10.1016/j.redox.2013.07.005 24024177 PMC3757712

[19] Rojo AI Sagarra MR Cuadrado A. GSK-3beta down-regulates the transcription factor Nrf2 after oxidant damage: relevance to exposure of neuronal cells to oxidative stress. J Neurochem. (2008) 105:192–202. 10.1111/j.1471-4159.2007.05124.x 18005231

[20] Cuadrado A Kugler S Lastres-Becker I. Pharmacological targeting of GSK-3 and NRF2 provides neuroprotection in a preclinical model of tauopathy. Redox Biol. (2018) 14:522–34. 10.1016/j.redox.2017.10.010 29121589 PMC5681345

[21] Rojo AI Rada P Egea J Rosa AO Lopez MG Cuadrado A. Functional interference between glycogen synthase kinase-3 beta and the transcription factor Nrf2 in protection against kainate-induced hippocampal cell death. Mol Cell Neurosci. (2008) 39:125–32. 10.1016/j.mcn.2008.06.007 18619545

[22] Salazar M Rojo AI Velasco D de Sagarra RM Cuadrado A. Glycogen synthase kinase-3beta inhibits the xenobiotic and antioxidant cell response by direct phosphorylation and nuclear exclusion of the transcription factor Nrf2. J Biol Chem. (2006) 281:14841–51. 10.1074/jbc.M513737200 16551619

[23] Chen H Zhang SM Hernan MA Schwarzschild MA Willett WC Colditz GA. Nonsteroidal anti-inflammatory drugs and the risk of Parkinson disease. Arch Neurol. (2003) 60:1059–64. 10.1001/archneur.60.8.1059 12925360

[24] Esposito E Di Matteo V Benigno A Pierucci M Crescimanno G Di Giovanni G. Non-steroidal anti-inflammatory drugs in Parkinson's disease. Exp Neurol. (2007) 205:295–312. 10.1016/j.expneurol.2007.02.008 17433296

[25] Moore AH Bigbee MJ Boynton GE Wakeham CM Rosenheim HM Staral CJ. Non-steroidal anti-inflammatory drugs in Alzheimer's disease and Parkinson's disease: reconsidering the role of neuroinflammation. Pharmaceuticals (2010) 3:1812–41. 10.3390/ph3061812 27713331 PMC4033954

[26] Tai W Ye X Bao X Zhao B Wang X Zhang D. Inhibition of Src tyrosine kinase activity by squamosamide derivative FLZ attenuates neuroinflammation in both *in vivo* and *in vitro* Parkinson's disease models. Neuropharmacology (2013) 75:201–12. 10.1016/j.neuropharm.2013.07.020 23916477

[27] Kim BW Koppula S Kumar H Park JY Kim IW More SV. alpha-Asarone attenuates microglia-mediated neuroinflammation by inhibiting NF kappa B activation and mitigates MPTP-induced behavioral deficits in a mouse model of Parkinson's disease. Neuropharmacology (2015) 97:46–57. 10.1016/j.neuropharm.2015.04.037 25983275

[28] Wang HL Gao JP Han YL Xu X Wu R Gao Y. Comparative studies of polydatin and resveratrol on mutual transformation and antioxidative effect *in vivo*. Phytomedicine (2015) 22:553–9. 10.1016/j.phymed.2015.03.014 25981921

[29] Liu H Zhao S Zhang Y Wu J Peng H Fan J. Reactive oxygen species-mediated endoplasmic reticulum stress and mitochondrial dysfunction contribute to polydatin-induced apoptosis in human nasopharyngeal carcinoma CNE cells. J Cell Biochem. (2011) 112:3695–703. 10.1002/jcb.23303 21815196

[30] Ji H Zhang X Du Y Liu H Li S Li L. Polydatin modulates inflammation by decreasing NF-kappaB activation and oxidative stress by increasing Gli1, Ptch1, SOD1 expression and ameliorates blood-brain barrier permeability for its neuroprotective effect in pMCAO rat brain. Brain Res Bull. (2012) 87:50–9. 10.1016/j.brainresbull.2011.09.021 22001340

[31] Chen Y Zhang DQ Liao Z Wang B Gong S Wang C. Anti-oxidant polydatin (piceid) protects against substantia nigral motor degeneration in multiple rodent models of Parkinson's disease. Mol Neurodegener. (2015) 10:4. 10.1186/1750-1326-10-4 26013581 PMC4506434

[32] Huang BX Liu JX Ma DX Chen GX Wang W Fu SP Myricetin prevents dopaminergic neurons from undergoing neuroinflammation-mediated degeneration in a lipopolysaccharide-induced Parkinson's disease model. J Funct Foods (2018) 45:452–461. 10.1016/j.jff.2018.04.018

[33] Monville C Torres EM Dunnett SB. Comparison of incremental and accelerating protocols of the rotarod test for the assessment of motor deficits in the 6-OHDA model. J Neurosci Methods (2006) 158:219–23. 10.1016/j.jneumeth.2006.06.001 16837051

[34] Iancu R Mohapel P Brundin P Paul G. Behavioral characterization of a unilateral 6-OHDA-lesion model of Parkinson's disease in mice. Behav Brain Res. (2005) 162:1–10. 10.1016/j.bbr.2005.02.023 15922062

[35] Haji Ghasem Kashani M Ghorbanian MT Hosseinpour L. Transplantation of deprenyl-induced tyrosine hydroxylase-positive cells improves 6-OHDA-lesion rat model of Parkinson's disease: behavioral and immunohistochemical evaluation. Cell J. (2013) 15:55–64. 23700561 PMC3660025

[36] Silva JM McMahon M The fastest Western in town: a contemporary twist on the classic Western blot analysis. J Vis Exp. (2014) 5:e51149 10.3791/51149 PMC4028330 24561642

[37] Anuranjani Bala M. Concerted action of Nrf2-ARE pathway, MRN complex, HMGB1 and inflammatory cytokines - implication in modification of radiation damage. Redox Biol. (2014) 2:832–46. 10.1016/j.redox.2014.02.008 25009785 PMC4085347

[38] Gupta SC Sundaram C Reuter S Aggarwal BB. Inhibiting NF-kappaB activation by small molecules as a therapeutic strategy. Biochim Biophys Acta (2010) 1799:775–87. 10.1016/j.bbagrm.2010.05.004 20493977 PMC2955987

[39] McCubrey JA Fitzgerald TL Yang LV Lertpiriyapong K Steelman LS Abrams SL. Roles of GSK-3 and microRNAs on epithelial mesenchymal transition and cancer stem cells. Oncotarget (2017) 8:14221–50. 10.18632/oncotarget.13991 27999207 PMC5355173

[40] McCubrey JA Steelman LS Bertrand FE Davis NM Sokolosky M Abrams SL. GSK-3 as potential target for therapeutic intervention in cancer. Oncotarget (2014) 5:2881–911. 10.18632/oncotarget.2037 24931005 PMC4102778

[41] Fakhoury M. Role of immunity and inflammation in the pathophysiology of neurodegenerative diseases. Neurodegener Dis. (2015) 15:63–9. 10.1159/000369933 25591815

[42] Hoban DB Connaughton E Connaughton C Hogan G Thornton C Mulcahy P. Further characterisation of the LPS model of Parkinson's disease: a comparison of intra-nigral and intra-striatal lipopolysaccharide administration on motor function, microgliosis and nigrostriatal neurodegeneration in the rat. Brain Behav Immun. (2013) 27:91–100. 10.1016/j.bbi.2012.10.001 23044176

[43] Michell-Robinson MA Touil H Healy LM Owen DR Durafourt BA Bar-Or A. Roles of microglia in brain development, tissue maintenance and repair. Brain (2015) 138(Pt 5):1138–59. 10.1093/brain/awv066 25823474 PMC5963417

[44] Dewapriya P Li YX Himaya SW Pangestuti R Kim SK. Neoechinulin a suppresses amyloid-beta oligomer-induced microglia activation and thereby protects PC-12 cells from inflammation-mediated toxicity. Neurotoxicology (2013) 35:30–40. 10.1016/j.neuro.2012.12.004 23261590

[45] Aloor R Zhang C Bandyopadhyay M Dasgupta S. Impact of nuclear factor-kappaB on restoration of neuron growth and differentiation in hippocampus of degenerative brain. J Neurosci Res. (2015) 93:1471–5. 10.1002/jnr.23547 25586448

[46] Prencipe G Minnone G Strippoli R De Pasquale L Petrini S Caiello I. Nerve growth factor downregulates inflammatory response in human monocytes through TrkA. J Immunol. (2014) 192:3345–54. 10.4049/jimmunol.1300825 24585880

[47] Vilhardt F Haslund-Vinding J Jaquet V McBean G. Microglia antioxidant systems and redox signalling. Br J Pharmacol. (2017) 174:1719–32. 10.1111/bph.13426 26754582 PMC5446583

[48] Jazwa A Cuadrado A. Targeting heme oxygenase-1 for neuroprotection and neuroinflammation in neurodegenerative diseases. Curr Drug Targets (2010) 11:1517–31. 10.2174/1389450111009011517 20704549

[49] Branca C Ferreira E Nguyen TV Doyle K Caccamo A Oddo S. Genetic reduction of Nrf2 exacerbates cognitive deficits in a mouse model of Alzheimer's disease. Hum Mol Genet. (2017) 26:4823–35. 10.1093/hmg/ddx361 29036636 PMC5886066

[50] Rojo AI Innamorato NG Martin-Moreno AM De Ceballos ML Yamamoto M Cuadrado A. Nrf2 regulates microglial dynamics and neuroinflammation in experimental Parkinson's disease. Glia (2010) 58:588–98. 10.1002/glia.20947 19908287

[51] Chen PC Vargas MR Pani AK Smeyne RJ Johnson DA Kan YW. Nrf2-mediated neuroprotection in the MPTP mouse model of Parkinson's disease: critical role for the astrocyte. Proc Natl Acad Sci USA. (2009) 106:2933–8. 10.1073/pnas.0813361106 19196989 PMC2650368

[52] Innamorato NG Lastres-Becker I Cuadrado A. Role of microglial redox balance in modulation of neuroinflammation. Curr Opin Neurol. (2009) 22:308–14. 10.1097/WCO.0b013e32832a3225 19359988

[53] Sandberg M Patil J D'Angelo B Weber SG Mallard C. NRF2-regulation in brain health and disease: implication of cerebral inflammation. Neuropharmacology (2014) 79:298–306. 10.1016/j.neuropharm.2013.11.004 24262633 PMC3958930

[54] Wang L Zhang S Cheng H Lv H Cheng G Ci X. Nrf2-mediated liver protection by esculentoside A against acetaminophen toxicity through the AMPK/Akt/GSK3beta pathway. Free Radic Biol Med. (2016) 101:401–12. 10.1016/j.freeradbiomed.2016.11.009 27836781

[55] Zhao Y Song W Wang Z Wang Z Jin X Xu J. Resveratrol attenuates testicular apoptosis in type 1 diabetic mice: role of Akt-mediated Nrf2 activation and p62-dependent Keap1 degradation. Redox Biol. (2018) 14:609–17. 10.1016/j.redox.2017.11.007 29154192 PMC5975057

[56] Xin Y Bai Y Jiang X Zhou S Wang Y Wintergerst KA. Sulforaphane prevents angiotensin II-induced cardiomyopathy by activation of Nrf2 via stimulating the Akt/GSK-3ss/Fyn pathway. Redox Biol. (2018) 15:405–17. 10.1016/j.redox.2017.12.016 29353218 PMC5975128

