# Supplementary material for: Polydatin Prevents Lipopolysaccharide (LPS)-Induced Parkinson's Disease via Regulation of the AKT/GSK3β-Nrf2/NF-κB Signaling Axis
Source: Front Immunol. 2018 Nov 5;9:2527. doi: 10.3389/fimmu.2018.02527 (PMC6230593; doi:10.3389/fimmu.2018.02527)
Supplement: Supplementary file 1 [file Data_Sheet_1.PDF]

Fig. S1

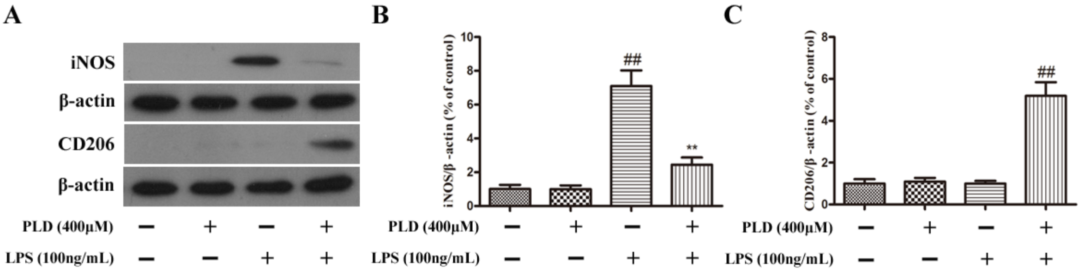

Fig. S1 Pretreatment with PLD suppressed M1 microglia phenotype and enhanced M2 microglia phenotype in LPS-treated BV-2 cells. After being pretreated with PLD (400 μM) for 1 h, BV-2 cells were treated with LPS (100 ng/mL) for 24 h. (A-C) the expression levels of iNOS and CD206 were measured by Western blotting. β-actin was utilized as an internal control. Similar results were obtained from three independent experiments. Values are presented as the mean ± SEM (n=4 in each group). $^{\#\#}p < 0.01$ vs. control group; $^{**}p < 0.01$ vs. LPS group.

Fig. S2

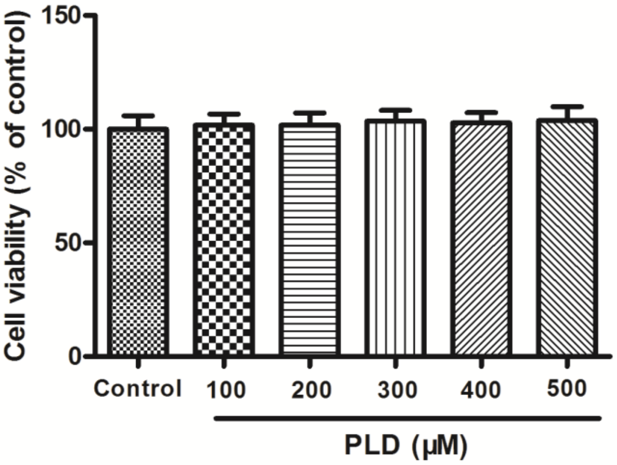

Fig. S2 Effects of PLD on the cell viability of BV-2 cells. BV-2 cells were stimulated with different concentrations of PLD for 24 h and then the cell viability was detected by the MTT assay. All experiments were repeated at least three times and similar results were observed. Values are mean ± SE, (n= 4 samples per group).
